# Early Exanthema Upon Vemurafenib Plus Cobimetinib Is Associated With a Favorable Treatment Outcome in Metastatic Melanoma: A Retrospective Multicenter DeCOG Study

**DOI:** 10.3389/fonc.2021.672172

**Published:** 2021-05-24

**Authors:** Katharina C. Kähler, Ralf Gutzmer, Friedegrund Meier, Lisa Zimmer, Markus Heppt, Anja Gesierich, Kai-Martin Thoms, Jochen Utikal, Jessica C. Hassel, Carmen Loquai, Claudia Pföhler, Lucie Heinzerling, Martin Kaatz, Daniela Göppner, Annette Pflugfelder, Ann-Sophie Bohne, Imke Satzger, Lydia Reinhardt, Jan-Malte Placke, Dirk Schadendorf, Selma Ugurel

**Affiliations:** ^1^ Department of Dermatology, University Hospital Schleswig-Holstein (UKSH), Kiel, Germany; ^2^ Department of Dermatology, University Hospital Hannover, Hannover, Germany; ^3^ Skin Cancer Center, National Center for Tumor Diseases, University Cancer Centre Dresden, Dresden, Germany; ^4^ Department of Dermatology, TU Dresden, University Hospital Carl Gustav Carus, Dresden, Germany; ^5^ Department of Dermatology, University Hospital Essen, German Cancer Consortium (DKTK), Essen, Germany; ^6^ Department of Dermatology, Universitätsklinikum Erlangen, Friedrich-Alexander University Erlangen-Nürnberg, Erlangen, Germany; ^7^ Department of Dermatology, University Hospital Würzburg, Würzburg, Germany; ^8^ Department of Dermatology, University Medical Center Göttingen, Göttingen, Germany; ^9^ Skin Cancer Unit, German Cancer Research Center (DKFZ), Heidelberg, Germany; ^10^ Department of Dermatology, Venereology and Allergology, University Medical Center Mannheim, Ruprecht-Karl University of Heidelberg, Mannheim, Germany; ^11^ Department of Dermatology, University Hospital Heidelberg, Heidelberg, Germany; ^12^ Department of Dermatology, University Hospital Mainz, Mainz, Germany; ^13^ Department of Dermatology, University Hospital Homburg, Homburg, Germany; ^14^ Department of Dermatology and Allergology, Ludwig-Maximilian University, München, Germany; ^15^ Department of Dermatology, SRH Waldklinikum, Gera, Germany; ^16^ Department of Dermatology, University Hospital Giessen, Gießen, Germany; ^17^ Department of Dermatology, University Hospital Tübingen, Tübingen, Germany

**Keywords:** melanoma, vemurafenib, cobimetinib, BRAF/MEK inhibition, skin toxicity, therapy outcome

## Abstract

**Background:**

The combination of BRAF and MEK inhibitors has become standard of care in the treatment of metastatic BRAF V600-mutated melanoma. Clinical factors for an early prediction of tumor response are rare. The present study investigated the association between the development of an early exanthema induced by vemurafenib or vemurafenib plus cobimetinib and therapy outcome.

**Methods:**

This multicenter retrospective study included patients with BRAF V600-mutated irresectable AJCC-v8 stage IIIC/D to IV metastatic melanoma who received treatment with vemurafenib (VEM) or vemurafenib plus cobimetinib (COBIVEM). The development of an early exanthema within six weeks after therapy start and its grading according to CTCAEv4.0 criteria was correlated to therapy outcome in terms of best overall response, progression-free (PFS), and overall survival (OS).

**Results:**

A total of 422 patients from 16 centers were included (VEM, n=299; COBIVEM, n=123). 20.4% of VEM and 43.1% of COBIVEM patients developed an early exanthema. In the VEM cohort, objective responders (CR/PR) more frequently presented with an early exanthema than non-responders (SD/PD); 59.0% versus 38.7%; p=0.0027. However, median PFS and OS did not differ between VEM patients with or without an early exanthema (PFS, 6.9 versus 6.0 months, p=0.65; OS, 11.0 versus 12.4 months, p=0.69). In the COBIVEM cohort, 66.0% of objective responders had an early exanthema compared to 54.3% of non-responders (p=0.031). Median survival times were significantly longer for patients who developed an early exanthema compared to patients who did not (PFS, 9.7 versus 5.6 months, p=0.013; OS, not reached versus 11.6 months, p=0.0061). COBIVEM patients with a mild early exanthema (CTCAEv4.0 grade 1-2) had a superior survival outcome as compared to COBIVEM patients with a severe (CTCAEv4.0 grade 3-4) or non early exanthema, respectively (p=0.047). This might be caused by the fact that 23.6% of patients with severe exanthema underwent a dose reduction or discontinuation of COBIVEM compared to only 8.9% of patients with mild exanthema.

**Conclusions:**

The development of an early exanthema within 6 weeks after treatment start indicates a favorable therapy outcome upon vemurafenib plus cobimetinib. Patients presenting with an early exanthema should therefore be treated with adequate supportive measures to provide that patients can stay on treatment.

## Introduction

Melanoma patients treated with BRAF and MEK inhibitors frequently develop an exanthema, also referred to as “skin rash” by non-dermatologists. This exanthema is typically characterized by inflammatory macules and papules but may also present with pustules or urticae. Its first signs commonly show within the first four to six weeks after therapy start. In the pivotal COBRIM trial the incidence of a skin rash upon monotherapy with vemurafenib was reported to be around 67.5% and during combination therapy with vemurafenib/cobimetinib the incidence was slightly higher with 72.5% (1). However, the term “skin rash” covers a variety of cutaneous side effects and thus cannot be equated with exanthema. Studies of EGFR inhibitors demonstrated an association of skin rash development with an improved therapy outcome in various cancer entities including colorectal carcinoma, head-and-neck squamous cell carcinoma, non-small cell lung cancer, prostate cancer, gastro-esophageal cancer, pancreatic adenocarcinoma and cutaneous squamous cell carcinoma (2, 3). Thus, in these cancer entities patients presenting with a skin rash under EGFR inhibitor therapy are encouraged to continue this treatment with the prospect of an increased probability of a favorable treatment outcome. For BRAF and MEK inhibition in metastatic melanoma, so far, no correlation has been reported between treatment efficacy and outcome and the occurrence of cutaneous side effects.

The present study was aimed to investigate the frequency and severity of an early exanthema upon BRAF and MEK inhibition with vemurafenib alone or combined with cobimetinib and its association with therapy outcome in patients with metastatic melanoma.

## Patients and Methods

This multicenter retrospective study was initiated by the Dermatologic Cooperative Oncology Group (DeCOG), and undertaken with Ethics Committee approval (Hannover University Medical School, 1612-2012). Patients were identified for study inclusion at clinical centers of the DeCOG based on the following eligibility criteria: histologically proven diagnosis of melanoma, unresectable metastatic disease in stage III or IV following the American Joint Committee on Cancer version 8 (AJCCv8) criteria (4), detection of a BRAF V600 mutation in the tumor tissue, treatment with vemurafenib as a single agent (VEM) or as the combination of cobimetinib plus vemurafenib (COBIVEM) within a time frame of June 01, 2012 and April 30, 2018, either as per clinical trial or *via* prescription, and availability of follow-up data after treatment start including adverse events, response and survival. The patients were identified at the centers *via* their digital hospital information systems or by chart review, and the requested data were extracted from the respective patient files.

### Data Collection

The requested data were collected on standardized electronic case report forms and merged in one central database for analysis. The data comprised patient demographics, BRAF V600 mutation subtype, sites of metastasis, overall performance status (OPS) graded by Eastern Cooperative Oncology Group (ECOG) criteria, and serum LDH activity, all at onset of VEM or COBIVEM therapy. For categorization of metastatic sites, we used the AJCCv8 M category by grouping by localization of metastases regardless of serum LDH activity. The used groups were (a) metastases to skin and/or lymph nodes (skin/LN), (b) metastases to the lung (lung), (c) metastases to other organs (other organs), and (d) metastases to the brain (brain). Data on other systemic treatments received by the patients before VEM or COBIVEM were recorded as previous treatments. This pre-treatment was categorized into (a) regimens containing immune checkpoint inhibitors (checkpoint inhibition), and (b) regimens containing kinase inhibitors (BRAF/MEK inhibition). Collected data on the course and outcome of VEM or COBIVEM therapy included therapy duration, best response following RECIST criteria (5) categorizing into complete response (CR), partial response (PR), stable disease (SD), and progressive disease (PD), as well as progression-free (PFS) and overall survival (OS). Patients were grouped into either objective responders (CR+PR) or non-responders (SD+PD). An exanthema presenting within the first six weeks after start of VEM or COBIVEM therapy was considered as an early exanthema, regardless of its morphology (macular, papular, pustular, urticae). The severity of the exanthema was graded according to CTCAEv4.0 (grade 1, <10% body surface area (BSA); grade 2, 10-30% BSA; grade 3, 30-100% BSA; grade 4, 100% BSA and/or severe reduction of general condition; grade 5, death) (6).

### Statistical Analysis

Data analysis was performed between January 01 and March 31, 2019. Survival (PFS, OS) was calculated from onset of VEM or COBIVEM until death or disease progression, respectively. If no such event occurred, the date of last patient contact was used as survival end point (censored observation). Survival curves, hazard ratios, and median survival times were calculated using the Kaplan–Meier method for censored failure time data. The log-rank test was used for comparison of survival probabilities between groups. Differences between groups were calculated using Fisher’s exact test or Chi square test. P<0.05 was considered statistically significant.

## Results

### Patient Characteristics and Early Exanthema

**Figure 1:**
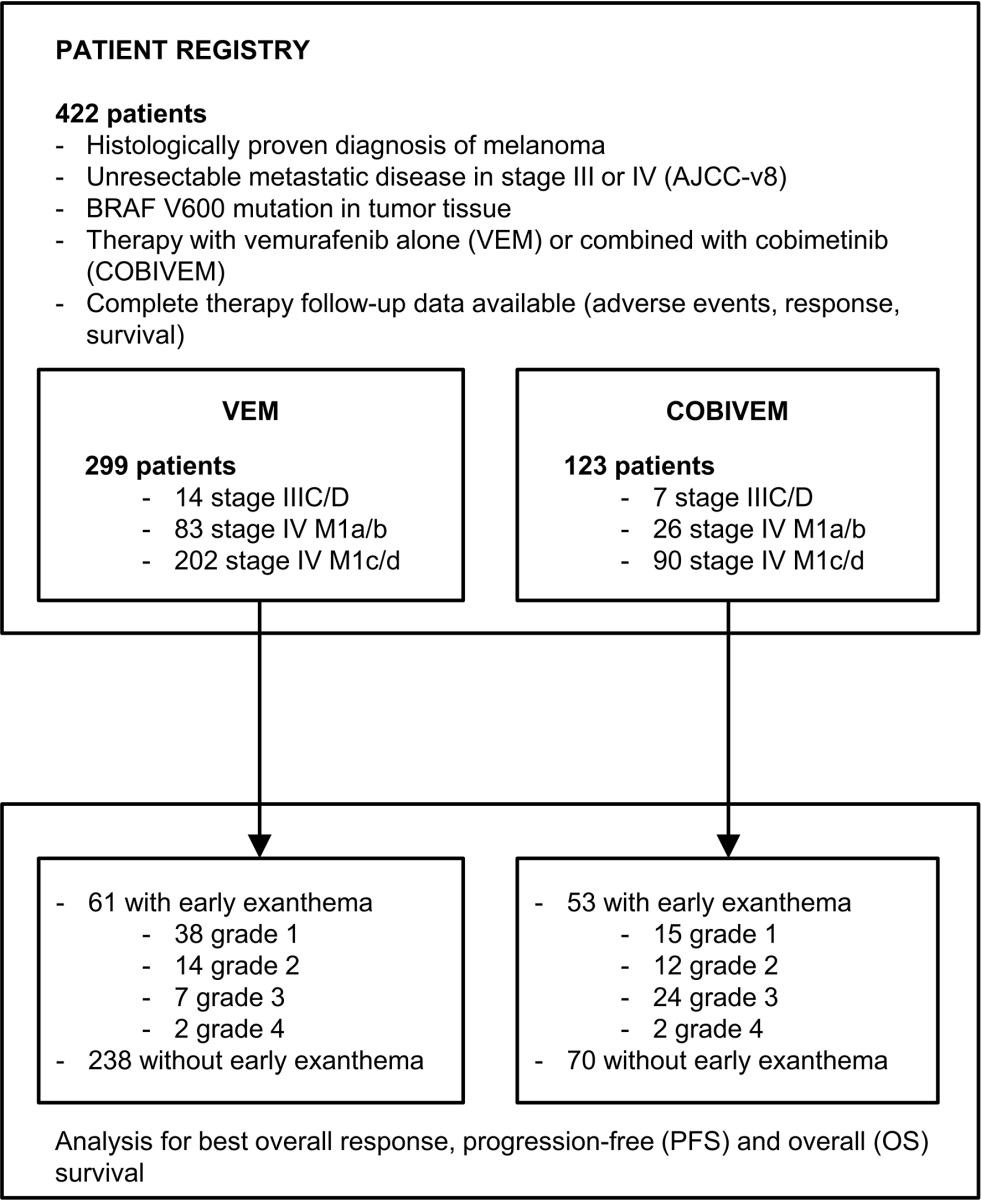
Schematic presentation of the study patient flow into patient registry. Patient inclusion criteria and grading of the early exanthemas was performed according to CTCAEv4.0 (grade 1, <10% body surface area (BSA); grade 2, 10-30% BSA; grade 3, 30-100% BSA; grade 4, 100% BSA and/or severe reduction of general condition).

**Figure 2:**
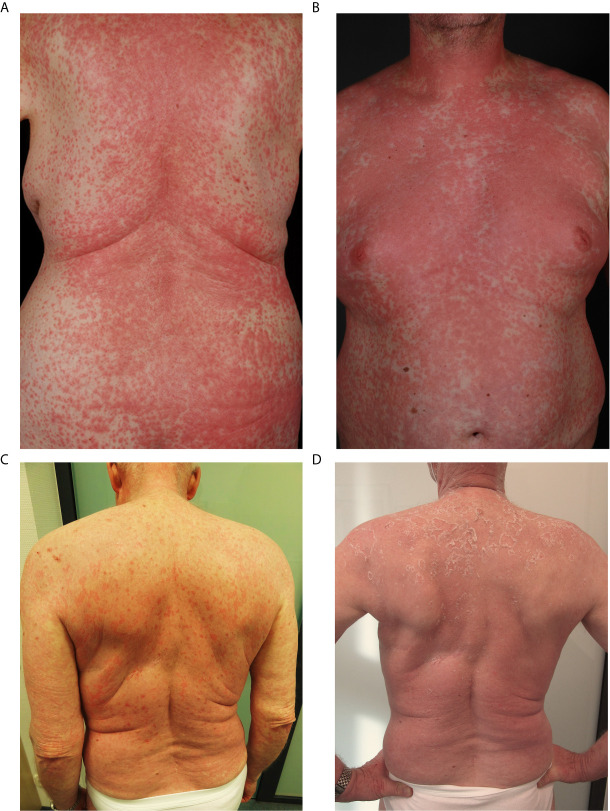
Representative patients from the study cohorts showing an early exanthema defined as onset within 6 weeks upon start of vemurafenib **(A)** or vemurafenib plus cobimetinib** (B)**, both grade 4 according to CTCAEv4.0. **(C)** Exanthem during vemurafenib and cobimetinib **(D)** follow-up after 4 weeks of topical and systemic steroids.

Data were collected of 422 patients at 16 clinical cancer centers in Germany. In total, 299 patients received VEM, 123 patients received COBIVEM. The patient flow is shown in **Figure 1**; detailed patient characteristics are presented in **Tables 1**, **2**. An early exanthema occurring within the first 6 weeks after start of therapy occurred in 61 VEM patients (20.4%) (CTCAE grade 1, 62.3%; grade 2, 22.9%; grade 3, 11.4%; and grade 4, 3.2%) and in 53 COBIVEM patients (43.1%) (CTCAE grade 1, 28.3%; grade 2, 22.6%; grade 3, 45.2%; and grade 4, 3.7%). Representative patients from both cohorts are demonstrated in **Figure 2**. In the VEM cohort, most patient characteristics at therapy start were balanced between groups with and without occurrence of an early exanthema, besides patients’ sex with females more often represented within the group of patients developing early exanthema than males (p=0.043; **Table 1**). In the COBIVEM cohort, the overall performance status at therapy start differed significantly between groups with and without occurrence of an early exanthema with patients presenting at ECOG 0 being strongly over-represented in the group developing an early exanthema (p=0.0058; **Table 2**). Age or LDH were not identified to be an influencing factor for the incidence of early exanthema (p= 0.11, **Table 2**).

**Table 1:** Patients treated with vemurafenib (VEM).

	Total n=299 (100%)	Early exanthema n=61 (100%)	No early exanthema n=238 (100%)	P-value	Relative risk
**Patient characteristics at therapy start**					
Sex					
male	164 (54.8%)	26 (42.6%)	138 (58.0%)		
female	135 (45.2%)	35 (57.4%)	100 (42.0%)	**0.043**	1.64
Age at treatment onset					
≤65 years	199 (66.6%)	39 (63.9%)	160 (67.2%)		
>65 years	100 (33.4%)	22 (36.1%)	78 (32.8%)	0.65	1.12
Localisation of primary					
skin	248 (82.9%)	50 (82.0%)	198 (83.2%)		
occult (MUP)	51 (17.1%)	11 (18.0%)	40 (16.8%)	0.85	1.07
Pre-treatment in stage III/IV					
no	169 (56.5%)	30 (49.2%)	139 (58.4%)		
yes	130 (43.5%)	31 (50.8%)	99 (41.6%)	0.25	1.34
BRAF/MEK inhibition	0 (0.0%)	0 (0.0%)	0 (0.0%)		
checkpoint inhibition	25 (8.4%)	6 (9.8%)	19 (8.0%)		
chemotherapy	127 (42.5%)	30 (49.2%)	87 (36.6%)		
Serum LDH					
normal (≤ULN)	150 (50.2%)	30 (49.2%)	120 (50.4%)		
elevated (>ULN)	149 (49.8%)	31 (50.8%)	118 (49.6%)	0.89	1.04
OPS (ECOG)					
0	177 (59.2%)	39 (63.9%)	138 (58.0%)		
≥1	110 (36.8%)	15 (24.6%)	95 (39.9%)	0.088	0.62
not specified	12 (4.0%)	7 (11.5%)	5 (2.1%)		
Stage (sites of metastasis)					
IIIC/D (skin/LN)	14 (4.7%)	8 (13.1%)	6 (2.5%)		
IV M1a (skin/LN)	46 (15.4%)	6 (9.8%)	40 (16.8%)		
IV M1b (lung)	37 (12.4%)	4 (6.6%)	33 (13.9%)		
IV M1c/d (other organ/brain)	202 (67.6%)	43 (70.5%)	159 (66.8%)	0.15	
BRAF V600 mutation status					
V600E	169 (56.5%)	34 (55.7%)	135 (56.7%)		
V600K	24 (8.0%)	5 (8.2%)	19 (8.0%)		
V600D	1 (0.3%)	0 (0.0%)	1 (0.4%)		
not further specified	105 (35.1%)	22 (36.1%)	83 (34.9%)	0.96	
**Therapy outcome**					
Best overall response					
CR	12 (4.0%)	3 (4.9%)	9 (3.8%)		
PR	161 (53.8%)	33 (54.1%)	128 (53.8%)		
SD	66 (22.1%)	15 (24.6%)	51 (21.4%)		
PD	47 (15.7%)	6 (9.8%)	41 (17.2%)		
NE	13 (4.3%)	4 (6.6%)	9 (3.8%)		
objective response (CR + PR)	128 (42.8%)	36 (59.0%)	92 (38.7%)	**0.0027**	**2.12**
Disease progression	207 (69.2%)	47 (77.0%)	160 (67.2%)		
Median PFS	6.3 months	6.9 months	6.0 months	0.65	HR=1.08
Death	144 (48.2%)	33 (54.1%)	111 (46.6%)		
Median OS	12.0 months	11.0 months	12.4 months	0.69	HR=1.09

The given patient characteristics refer to the start of vemurafenib (VEM) therapy. Percentages are given per column. Stage categories refer to the AJCCv8 classification system. Pre-treatment describes systemic therapies received by the patient for inoperable stage III or IV disease (non-adjuvant) prior to VEM therapy. Patient groups with and without early exanthema were compared by Fisher’s exact test or Chi square test; results are given by p-values, relative risks or hazard ratios. MUP, melanoma of unknown primary; LDH, lactate dehydrogenase; ULN, upper limit of normal; OPS, overall performance status; CR, complete response; PR, partial response; SD, stable disease; PD, progressive disease; NE, not evaluable. Bold means statistically significant.

**Table 2:** Patients treated with cobimetinib plus vemurafenib (COBIVEM).

	Total n=123 (100%)	Early exanthema n=53 (100%)	No early exanthema n=70 (100%)	P-value	Relative risk
**Patient characteristics at therapy start**					
Sex					
male	69 (56.1%)	27 (50.9%)	42 (60.0%)		
female	54 (43.9%)	26 (49.1%)	28 (40.0%)	0.36	1.23
Age at treatment onset					
≤65 years	88 (71.5%)	42 (79.2%)	46 (65.7%)		
>65 years	35 (28.5%)	11 (20.8%)	24 (34.3%)	0.11	0.66
Localisation of primary					
skin	108 (87.8%)	47 (88.7%)	61 (87.1%)		
occult (MUP)	15 (12.2%)	6 (11.3%)	9 (12.9%)	1.0	0.92
Pre-treatment in stage III/IV					
no	55 (44.7%)	24 (45.3%)	31 (44.3%)		
yes	68 (55.3%)	29 (54.7%)	39 (55.7%)	1.0	0.98
BRAF/MEK inhibition	43 (34.9%)	12 (22.6%)	31 (44.3%)		
checkpoint inhibition	44 (35.8%)	17 (32.1%)	27 (38.6%)	0.36	1.38
Serum LDH					
normal (≤ULN)	72 (58.5%)	31 (58.5%)	41 (58.6%)		
elevated (>ULN)	51 (41.5%)	22 (41.5%)	29 (41.4%)	1.0	1.0
OPS (ECOG)					
0	83 (67.5%)	42 (79.2%)	41 (58.6%)		
≥1	38 (30.9%)	9 (17.0%)	29 (41.4%)	**0.0058**	0.47
not specified	2 (1.6%)	2 (3.8%)	0 (0.0%)		
Stage (sites of metastasis)					
IIIC/D (skin/LN)	7 (5.7%)	1 (1.9%)	6 (8.6%)		
IV M1a (skin/LN)	13 (10.6%)	7 (13.2%)	6 (8.6%)		
IV M1b (lung)	13 (10.6%)	8 (15.1%)	5 (7.1%)		
IV M1c/d (other organ/brain)	90 (73.1%)	37 (69.8%)	53 (75.7%)	0.18	
BRAF V600 mutation status					
V600E	92 (74.8%)	39 (73.6%)	53 (75.7%)		
V600K	15 (12.2%)	6 (11.3%)	9 (12.9%)		
V600R	2 (1.6%)	1 (1.9%)	1 (1.4%)		
V600D	1 (0.8%)	1 (1.9%)	0 (0.0%)		
K601E	1 (0.8%)	0 (0.0%)	1 (1.4%)		
not further specified	12 (9.8%)	6 (11.3%)	6 (8.6%)	0.79	
**Therapy outcome**					
Best overall response					
CR	13 (10.6%)	8 (15.1%)	5 (7.1%)		
PR	60 (48.8%)	27 (50.9%)	33 (47.1%)		
SD	23 (18.7%)	8 (15.1%)	15 (21.4%)		
PD	18 (14.6%)	3 (5.7%)	15 (21.4%)		
NE	9 (7.3%)	7 (13.2%)	2 (2.9%)		
objective response (CR + PR)	73 (59.3%)	35 (66.0%)	38 (54.3%)	**0.031**	1.79
Disease progression	77 (62.6%)	30 (56.6%)	47 (67.1%)		
Median PFS	7.3 months	9.7 months	5.6 months	**0.013**	HR=0.55
Death	37 (30.1%)	7 (13.2%)	30 (42.9%)		
Median OS	not reached	not reached	11.6 months	**0.0061**	HR=0.39

The given patient characteristics refer to the start of cobimetinib plus vemurafenib (COBIVEM) therapy. Percentages are given per column. Stage categories refer to the AJCCv8 classification system. Pre-treatment describes systemic therapies received by the patient for inoperable stage III or IV disease (non-adjuvant) prior to COBIVEM therapy. Patient groups with and without early exanthema were compared by Fisher’s exact test or Chi square test; results are given by p-values, relative risks or hazard ratios. MUP, melanoma of unknown primary; LDH, lactate dehydrogenase; ULN, upper limit of normal; OPS, overall performance status; CR, complete response; PR, partial response; SD, stable disease; PD, progressive disease; NE, not evaluable. Bold means statistically significant.

### VEM and COBIVEM Therapy and Outcome

All patients started with the initial doses of 960 mg vemurafenib orally b.i.d. (VEM) or vemurafenib 960 mg orally b.i.d. plus cobimetinib 60 mg orally once daily (COBIVEM). Due to the occurrence of an early exanthema, 32.7% of VEM patients and 26.8% of COBIVEM patients had a dose reduction, and 11.4% of VEM and 5.7% of COBIVEM patients had a therapy discontinuation. At database closure on September 30, 2019, the median follow-up time was 21.6 months. 48.2% of the VEM patients and 30.1% of the COBIVEM patients had died. Of the patients alive, 27.4% were still on VEM treatment, and 30.8% on COBIVEM treatment.

As best overall response, 4.0% of VEM patients achieved a CR, 53.8% achieved a PR, 22.1% showed a SD, and 15.7% revealed a disease progression. 4.3% of the patients were not evaluable for treatment response due to other reasons. Patients presenting an early exanthema upon VEM revealed a superior therapy response with an objective response rate (CR+PR) of 59.0% in patients showing an early exanthema versus 38.7% in patients without this cutaneous reaction (p=0.0027; **Table 1**). In the patient cohort treated with COBIVEM, 10.6% of patients achieved a CR, 48.8% achieved a PR, 18.7% showed a SD, and 14.6% revealed disease progression. 7.3% of the patients were not evaluable for therapy response. Here again, patients showing an early exanthema upon treatment had a higher objective response rate than patients who did not (66.0% versus 54.3%; p=0.031; **Table 2**).

**Figure 3:**
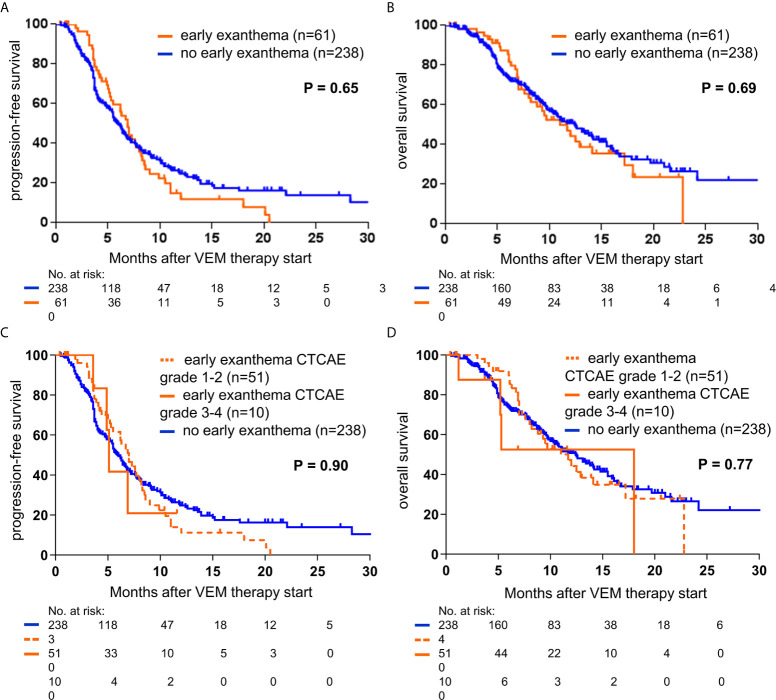
Kaplan-Meier curves showing the probability of progression-free **(A, C)**, and overall survival **(B, D)**, of metastatic melanoma patients treated with vemurafenib (VEM; n=299). Survival curves are displayed for patients with or without presentation of early exanthema upon treatment. Censored observations are indicated by vertical bars. P-values were calculated using the log rank test.

**Figure 4:**
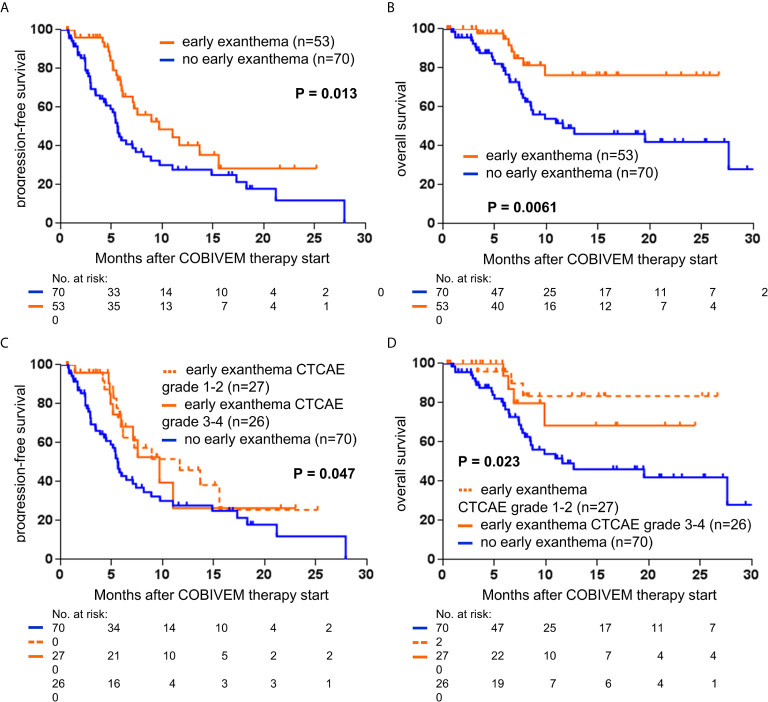
Kaplan-Meier curves showing the probability of progression-free **(A, C)** and overall survival **(B, D)** of metastatic melanoma patients treated with vemurafenib plus cobimetinib (COBIVEM; n=123). Survival curves are displayed for patients with or without presentation of early exanthema upon treatment. Censored observations are indicated by vertical bars. P-values were calculated using the log rank test.

With regard to survival after therapy start, for patients treated with VEM median PFS and OS were not significantly different for patients with or without an early exanthema (6.9 versus 6.0 months, p=0.65; 11.0 versus 12.4 months, p=0.69 respectively, **Figures 3A, B**). Additionally, the respective Kaplan-Meier survival curves were almost identical in shape and were crossing each other repeatedly. In contrast, for patients treated with COBIVEM survival after therapy start was significantly better in patients presenting an early exanthema. Median PFS and OS were significantly prolonged in patients showing an early exanthema versus patients who did not (PFS, 9.7 versus 5.6 months, p=0.013; OS, not reached versus 11.6 months, p=0.0061; **Figures 4A, B**). With regard to the severity of the early exanthema, patients who developed a mild exanthema (CTCAE grade 1-2) had a superior outcome in terms of PFS and OS compared to patients who developed a severe (CTCAE grade 3-4) exanthema or patients who developed no exanthema (p=0.047, **Figures 4C, D**).

## Discussion

Vemurafenib is a selective inhibitor of V600-mutated BRAF, and was the first-in-class mitogen-activated protein (MAP) kinase pathway inhibitor approved for the treatment of melanoma (7). Subsequently, the combination therapy of vemurafenib together with the MEK inhibitor cobimetinib was approved for metastatic melanoma due to the significant prolongation of survival times shown by clinical trial data (1, 8). Nevertheless, predictive markers of the treatment outcome of either vemurafenib monotherapy or vemurafenib plus cobimetinib combination therapy are rare and most often characterized by low specificity. Elevated serum LDH, as well as multiple organ involvement by metastases were shown to be associated with a less favorable treatment outcome of BRAF/MEK inhibition (9). However, these parameters are likewise associated with a poor treatment outcome upon immune checkpoint inhibition (10). Thus, other biomarkers associated with treatment outcome are urgently required to indicate a patient’s individual probability to benefit from vemurafenib/cobimetinib therapy. Optimally, these markers are detectable immediately before treatment start. However, biomarkers which become evident shortly after treatment start like cutaneous adverse events may also be of great help.

So far, only one retrospective analysis showed a possible correlation between the cutaneous side effects panniculitis and vitiligo-like lesions and the treatment outcome upon the BRAF plus MEK inhibitor combination dabrafenib and trametinib (11). Another retrospective case series showed a correlation between different cutaneous and extra-cutaneous adverse events including vitiligo, erythema nodosum, uveitis and keratitis sicca and the treatment outcome upon BRAF inhibitors either administered alone or in combination with MEK inhibitors (12). However, all these adverse events were reported in patients under BRAF/MEK inhibition, but at low frequencies and thus are of little use as predictive markers of treatment response in the majority of patients treated with BRAF/MEK inhibitors.

In contrast, exanthema is a common adverse event in patients treated with BRAF/MEK inhibitors (13). In clinical trials, 15.7% of patients treated with encorafenib/binimetinib developed a low grade rash/maculopapular rash (high grade 1%). Additional 3.1% showed an acneiform exanthem (high grade 0%). 27.7% of patients treated with dabarafenib/trametinib developed a low grade rash/maculopapular rash (high grade 1.5%). Additional 6.6% showed an acneiform exanthema (high grade 0%). The combination of vemurafenib/cobimetinib induced in 56.3% of patients a low grade rash/maculopapular rash (high grade 12.6%). Additional 13.8% showed an acneiform exanthema (high grade 2.4%). Important to acknowledge is the fact, that non-dermatologists do not differentiate between the common term rash and the specific characteristics of e.g. a maculopapular exanthema or acneiform exanthema (13). Additionally, in clinical trials the onset of exanthema is not specified, so the reported incidence of exanthema does not give further information about the rate of early exanthemas within the first weeks of treatment initiation. Moreover, an exanthema develops early during treatment, most often within the first four to six weeks of treatment, and is easily detectable by an inspection of the patient’s skin (13). These advantages render the detection of an early exanthema as a useful indicator of a favorable treatment outcome.

Interestingly, in the VEM cohort, females were more often represented within the group of patients developing early exanthema than males (p=0.043; **Table 1**). This has also been demonstrated to be a known risk factor for rash induced by BRAF/MEK inhibitors in the metaanalysis of Hopkins et al. (14).

This early exanthema is usually treated by a dose reduction of the BRAF/MEK inhibitors in combination with topical steroids and only in rare, severe cases with systemic steroids. Due to their early exanthema, 32.7% of VEM patients and 26.8% of COBIVEM patients needed a dose reduction.

Indeed, in our study we found that the occurrence of an exanthema within the first six weeks of treatment was significantly associated with an improved response rate and a prolonged survival in terms of PFS and OS in patients treated with COBIVEM. In patients treated with VEM, the development of an early exanthema was correlated with an improved objective response, but did not show an association to an improved survival.

Possible reasons for this differential impact on survival remain to be elucidated. First it should be mentioned that the early exanthema during COBIVEM and other BRAF/MEK combination therapies has to be differentiated from the acneiform rash induced specifically by MEK inhibitor monotherapies. This acneiform rash commonly occurs later during treatment, most often between week 6 and 12 after treatment start, and has a well-defined causal mechanism (13). The early exanthema developing within the first six weeks of COBIVEM treatment might be induced by the immune activation described for MEK inhibition therapies. It has been demonstrated that COBIVEM as well as dabrafenib plus trametinib therapy induces a type I interferon response in keratinocytes which acts proinflammatory and antineoplastically (15). In histopathology analysis, a slight basal layer vacuolization, dermal edema and a superficial dermal perivascular lymphocyte and eosinophil infiltrate was described (16). Also, it has been demonstrated that a pre-treatment with MEK inhibitors enhances immune responses, tumor-infiltrating T cells, and an immune-stimulating tumor microenvironment (17).

Interestingly, patients developing a mild exanthema revealed a stronger benefit from COBIVEM therapy than patients with a severe exanthema or patients without any exanthema. This finding might be explained by the fact that of the patients who developed a severe exanthema, 18.7% underwent a dose reduction of COBIVEM and 4.9% completely discontinued the treatment, compared to only 8.1% of patients who developed a mild exanthema that needed a dose reduction and 0.8% that discontinued the treatment. In contrast, it has been shown that dose reductions of BRAF/MEK inhibitors due to early toxicity in the first 28 days are significantly associated with improved survival, progression free survival and response (18, 19). However, following our present results, patients developing an early exanthema upon COBIVEM are patients with a high probability of a favorable therapy outcome and should thus be supported to continue treatment with COBIVEM. This support can be provided by an adequate therapeutic management of the exanthema, e.g. by the use of topical corticosteroids and/or anti-pruritics.

In conclusion, our results indicate that the development of an early exanthema upon BRAF/MEK inhibition with COBIVEM is a surrogate marker of a favorable therapy outcome in metastatic melanoma patients. Thus, patients presenting with an early exanthema under COBIVEM therapy should be treated with adequate supportive measures to provide that patients can stay on treatment. As a limitation, our findings result from a retrospective analysis and should therefore be confirmed in prospective clinical trials or registries.

## Data Availability Statement

The original contributions presented in the study are included in the article/supplementary material. Further inquiries can be directed to the corresponding author.

## Ethics Statement

The studies involving human participants were reviewed and approved by Ethics Committee approval (Hannover University Medical School, 1612-2012). Written informed consent for participation was not required for this study in accordance with the national legislation and the institutional requirements.

## Author Contributions

SU and KK contributed to conception and design of the study. All authors contributed to the acquisition pf data. SU organized the database. SU and KK performed the statistical analysis. KK wrote the first draft of the manuscript. SU and KK wrote sections of the manuscript. All authors contributed to the article and approved the submitted version.

## Funding

This study was performed within the network of study centers of the German Dermatologic Cooperative Oncology Group (DeCOG). This research did not receive any specific grant from funding agencies in the public, commercial, or not-for-profit sectors.

## Conflict of Interest

KK has served as consultant or/and has received honoraria from Amgen, Roche, Bristol Myers Squibb, Merck Sharp and Dohme, Pierre Fabre, and Novartis, and received travel support from Amgen, Merck Sharp and Dohme, Bristol Myers Squibb, Amgen, Pierre Fabre, Medac, and Novartis. RG received honoraria for lectures and advisory boards, research support and meeting support from Almirall Hermal, Amgen, Astra Zeneca, Bristol Myers Squibb, Leo, Merck Serono, Merck Sharp and Dohme, Pierre Fabre, Roche, Sanofi Genzyme, Regeneron, Sun Pharma, Takeda, Pfizer, Novartis, Johnson&Johnson, 4SC, and Incyte. FM has received travel support or/and speaker’s fees or/and advisor’s honoraria by Novartis, Roche, BMS, MSD and Pierre Fabre, and research funding from Novartis and Roche. LZ has served as consultant and/or has received honoraria from Roche, Bristol Myers Squibb, Merck Sharp and Dohme, Novartis, Pierre Fabre, and Sanofi, and received travel support from Bristol Myers Squibb, Merck Sharp and Dohme, Amgen, Pierre Fabre, and Novartis. MH has received consultant and/or speaker honoraria form Bristol Myers Squibb, Novartis, Merck Sharp and Dohme, Sanofi, Roche and travel support from Novartis, and Bristol Myers Squibb. AG reports speakers honoraria from Bristol Myers Squibb, Merck Sharp and Dohme, and Roche, advisory board honoraria from Bristol Myers Squibb, Novartis, Merck Sharp and Dohme, Pierre Fabre, Pfizer, Roche and Sanofi Genzyme, and travel support from Bristol Myers Squibb, Merck Sharp and Dohme, Novartis, and Roche. K-MT received honoraria for lectures and advisory boards from Bristol-Myers Squibb, Roche, Novartis, Merck Sharp and Dohme, Pierre Fabre, LEO, Galderma, AbbVie, La Roche-Posay and Candela, and travel support from Bristol-Myers Squibb, Roche, Novartis, Merck Sharp and Dohme, Pierre Fabre, LEO, and Candela. JU is on the advisory board or has received honoraria and travel support from Amgen, Bristol Myers Squibb, GSK, LeoPharma, Merck Sharp and Dohme, Novartis, Pierre Fabre, Roche, Sanofi outside the submitted work. JH reports speakers honoraria from Bristol Myers Squibb, Novartis, Merck Sharp and Dohme, and Roche, advisory board honoraria from Merck Sharp and Dohme, Pierre Fabre, Sunpharma and Sanofi Genzyme, and travel support from Bristol Myers Squibb, and Pierre Fabre. CL declares speakers and advisory board honoraria and travel support from Bristol Myers Squibb, Merck Sharp and Dohme, Merck Serono, Novartis, Roche, Amgen, Pierre Fabre, and Sun Pharma. CP received speaker or consultant honoraria and travel support from Novartis, Bristol Myers Squibb, Roche, Merck Serono, Merck Sharp and Dohme, Celgene, AbbVie, and LEO. LH received grants from Novartis, and has received speaker or consultant fees personal fees from Amgen, Bristol Myers Squibb, Merck Sharp and Dohme, Roche, Curevac, Pierre Fabre, Roche, Novartis, and Sanofi. MK has received grants from Bristol Myers Squibb, Merck Sharp and Dohme, Leo, Novartis, and Roche.

DG declares speakers and advisory honoraria as well as travel support from Bristol Myers Squibb, Novartis, Pierre Fabre, Sanofi Genzyme, Amgen, Galderma, Janssen, and Roche.

DS declares advisory board and speakers honoraria from Roche, Novartis, Bristol-Myers-Squibb, MSD, Merck-Serono, Sanofi, Nektar, Amgen, Hexal, InFlaRx, Array, Pierre Fabre, Immunocore, Philogen Sun Pharma, Regeneron, and Ultimovacs, as well as grant and travel support from Roche, Novartis, Bristol-Myers-Squibb, MSD, Merck-Serono, and Sanofi. SU declares research support from Bristol Myers Squibb, and Merck Serono, speakers and advisory board honoraria from Bristol Myers Squibb, Merck Sharp and Dohme, Merck Serono, Novartis and Roche, and travel support from Bristol Myers Squibb, Merck Sharp, and Dohme.

The remaining authors declare that the research was conducted in the absence of any commercial or financial relationships that could be construed as a potential conflict of interest.
